# Comparative analysis of asbestos body and fiber content in formalin-fixed vs. paraffin-embedded lung tissue

**DOI:** 10.3389/fpubh.2025.1590802

**Published:** 2025-06-11

**Authors:** Barbara K. Kuhn, Bart Vrugt, Alex Low, Julie E. Goodman, Richard Attanoos

**Affiliations:** ^1^Silag – Swiss Laboratory for Particle Analysis in Tissues, Zurich, Switzerland; ^2^Institute of Pathology, Stadtspital Triemli, Zurich, Switzerland; ^3^Gradient, Boston, MA, United States; ^4^Department of Cellular Pathology and School of Medicine Cardiff University, University Hospital of Wales, Wales, United Kingdom

**Keywords:** asbestos bodies, amphibole asbestos fibers, formalin-fixed lung tissue, paraffin-embedded tissue, paraffin block

## Abstract

**Introduction:**

Asbestos body and fiber burdens may be determined using different preparations of lung tissue. Paraffin-embedded tissue requires more complex steps than formalin-fixed tissue. A prior study highlighted potential variations in the measurement of retained mineral fibers in different lung preparations and prompted this expanded interlaboratory analysis. Data from exposed subjects referred to a Swiss laboratory were compared with the results of mineral analysis obtained from a laboratory based in the United Kingdom.

**Methods:**

Numbers of asbestos bodies were determined in formalin-fixed tissue and corresponding paraffin blocks of 62 subjects in Zurich by NaOCl digestion. Fiber burden was measured in a total of 104 subjects (62 subjects in Zurich and 42 subjects in Cardiff).

**Results:**

Asbestos body and amphibole asbestos fiber counts obtained from paraffin blocks were noted to be, in general, lower than counts obtained from formalin-fixed tissue. The limits of detection were higher in paraffin blocks than in formalin-fixed tissue. Similar trends were obtained in the two laboratories.

**Discussion:**

In this comparative mineral analytic study, the authors focused on the potential significance of differing specimen preparations (formalin-fixed wet lung versus paraffin wax-embedded block extraction) investigating paired samples. The results generally reflect numerically higher fiber burdens in samples analyzed from wet lungs compared with counterpart paraffin wax tissue. Mineral analysis by electron microscopic analysis remains the most objective measure of the respirable fraction of mineral dust as it correlates most directly with disease risk.

## 1 Introduction

Asbestos has been associated with the development of mesothelioma, lung cancer, lung fibrosis (asbestosis), and benign pleural disease. Despite bans in industrialized countries, asbestos exposure is still a global health hazard due to its biopersistence, the long latency periods of asbestos-related diseases, the ongoing asbestos use in developing countries, and the large legacy from past use in industrialized countries (1). The strength of the association between asbestos and its diseases is governed by cumulative dose, fiber type, fiber size, and fiber durability (2–4).

Asbestos is a collective term for six regulated naturally occurring, fibrous silicate minerals from two groups: serpentine and amphibole. These include serpentine group mineral chrysotile and amphibole types, crocidolite (riebeckite asbestos), amosite (cummingtonite-grunerite asbestos), anthophyllite asbestos, tremolite asbestos, and actinolite asbestos. Assessing an individual’s prior asbestos exposure is problematic and may be performed using clinical history or hygienists’ dose reconstructions (both of which rely on reported exposures), or pathologic-mineralogic grounds. Mineral fiber analysis has been shown to be a reliable arbiter for assessing an individual’s prior exposure. It is the only exposure assessment method that can determine an individual’s respirable dose to specific minerals and their dimensions and benchmark the same against known control populations, to contextualize the significance of the individual exposure to disease.

Mineral analysis is an established method for determining retained asbestos and other mineral content in the lungs of people seeking compensation for asbestos-related disease—especially mesothelioma, lung fibrosis/asbestosis, and lung cancer (5–8). It is recognized that anatomic variations in fiber concentrations exist, so three samples of ~2 cm^3^ non-involved lung tissue from different anatomic sites are generally required to yield representative results commensurate with prior exposures (6, 8, 9). Typically, this is best achieved from autopsy material or pneumonectomy specimens. Samples obtained from lobectomy or open lung biopsies may be acceptable for fiber quantification, but caution is warranted, as wider tissue sampling is not possible. The ERS Taskforce guidelines for mineral analysis on biologic samples (6) proposed sampling strategies and preparation with a focus on formalin-fixed tissue, but if samples are representative, it is acceptable to use paraffin blocks for analysis. As the technical procedures for utilizing paraffin-embedded material and removal include a considerable number of additional washing steps during which mineral content can potentially be lost, assessing the comparability of results between the two materials is important, particularly because there has been a shift toward using paraffin-embedded materials for mineral analysis. However, there is a limited amount of data with respect to asbestos content in formalin-fixed and paraffin-embedded tissue from the same subject. Roggli et al. (10) determined a weight loss in paraffin block and suggested a correction factor of 0.7 if results are given per gram of wet tissue. Kuhn et al. (11) also evaluated a comparative analysis, which we expanded upon, focusing on asbestos body (AB) and asbestos fiber count yields in formalin-fixed and paraffin-embedded lung tissue. This study was conducted in two European laboratories and includes the 37 cases from Kuhn et al. (11) and an additional 67 cases (25 Swiss-Zurich and 42 UK-Cardiff), for a total of 104 subjects.

## 2 Patients and methods

### 2.1 Analysis in Zurich

#### 2.1.1 Sample collection

Formalin-fixed lung tissue (FF) and corresponding paraffin-embedded tissue (block, PE) from 25 patients (9 with asbestosis, 10 with mesothelioma, and 6 with lung carcinoma) were retrieved from the archive of Silag. Combined with the data reported by Kuhn et al. (11), this adds up to 62 patients. From 9 subjects, samples from several lung lobes were available and prepared separately for analysis. Therefore, we analyzed 75 sample pairs in Zurich. The Cardiff data are described in Section 2.2.

Mineral fiber analysis methods have been developed and published elsewhere (12). Formalin-fixed tissue was cut to take samples for paraffin-embedding and histology. One paraffin block was selected for analysis. Tissue from paraffin blocks was retrieved by cutting away overstanding free paraffin and by washing the embedded tissue in Histoclear (stirring for 1 h) three times. Subsequently, the tissue was washed in 100% pure ethanol (stirring for 1 h) three times. After that, both formalin-fixed and retrieved tissues were split.

#### 2.1.2 Digestion for AB counting

Four times 1 g of formalin-fixed tissue and one-third of the paraffin-embedded tissue were digested in NaOCl. After complete digestion, adequate concentrations were filtered on 1.2 μm mixed cellulose filters, and the complete filter was screened for asbestos bodies at 100X. The average +/− standard deviation from the 4 readings in formalin-fixed tissue is given as AB / g dry tissue in FF. For PE, two 50% filters were made and the average +/− standard deviation is given as AB content.

#### 2.1.3 Low-temperature ashing for fiber counting

In total, 5 g of FF or two-thirds of PE were freeze-dried overnight and low-T ashed until Δm < 0.5 mg. The ash was taken up into 5 mL HCl (2 M) + 20 mL H_2_O. After 10 s in the ultrasonic bath, the solution was stirred for 30 min and then centrifuged at 340 rpm (2,100 g) for 20 min. The supernatant was decanted and replaced by H_2_O. After 10 s in the ultrasonic bath, the sample was stirred for 15 min followed by centrifugation at 340 rpm (2,100 g) for 20 min. The supernatant was decanted and replaced by 100% ethanol. After 10 s in the ultrasonic bath, the sample was stirred for 15 min and centrifuged for a third time at 340 rpm (2,100 g) for 20 min. The supernatant was decanted and replaced by H_2_O. Two drops of 0.1% triton were added to the sample. After 10 s in the ultrasonic bath and being stirred for 15 min, the solution was filtered in adequate concentration on 0.2 μm polycarbonate filters. The filters were coated with 10 nm of Carbon. Jaffe transfer was applied to get the sample on Cu TEM grids (75×300 mesh).

#### 2.1.4 Electron microscopy analysis

Fiber analysis was done using a FEI transmission electron microscope (TEM) equipped with an energy-dispersive X-ray spectrometer (EDX). Acceleration voltage was 120 kV, and magnification of 4,200X was used for fiber counting and 12,000–17,000X for fiber identification and dimension measurements. All fibers (l > 0.5 μm; parallel sides; aspect ratio ≥ 3:1) on 25 grid fields were counted, and the first 25 fibers were analyzed. Total asbestos and amphibole asbestos fiber (AAF) content was calculated separately per gram of dry tissue.

### 2.2 Analysis in Cardiff

Mineral fiber analysis methods have been developed and published elsewhere (13). Fiber analysis was performed on formalin-fixed or paraffin-embedded lung tissue specimens. Mineral analysis was conducted on ‘pooled’ lung samples obtained from optimally three lung regions: lung upper lobe, apex lower lobe, and lung base (6, 9). AB counting was not performed in this laboratory.

#### 2.2.1 Digestion and ashing for fiber counting

Paraffin-embedded tissue blocks were melted down in hot molten wax, labeled, and heated at 100°C for 15–20 min and then 60°C for 30 min. Xylene was added and underwent seven changes over approximately 26 h, followed by three changes into 100% IDA each 30 min and one change in 70% IDA for 30 min. The sample was then placed into distilled water prior to digestion in potassium hydroxide. Formalin-fixed “wet” tissue was directly digested in 40% potassium hydroxide.

Following digestion, the sample was washed with distilled water and then centrifuged at 4000 rpm for 20 min. This was repeated at least three times, with sequential removal of the supernatant and replacement with distilled water until the pH was 7. The last supernatant was removed, and the residue was placed in a 350°C heater block with oxygen and left overnight to ash. Then, 10 mL of 0.3 M HCl was added to the ashed residue and mixed in an ultrasonic water bath for a minimum of 2 min. The residue was transferred to a nucleopore membrane, starting at 20%, filtered, then carbon coated, and was ready for sections to be taken with gold TEM grids.

#### 2.2.2 Electron microscopy analysis

Cases were examined with a Phillips transmission electron microscope (TEM) equipped with an energy dispersive x-ray analyzer (EDX). A low-power evaluation (1,000X) of the grid was performed to ensure sample uniformity. Elongate structures identified at 22000X and 80 KV measuring >0.5 μm in length, with parallel sides and an aspect ratio of at least 3:1, were measured in two dimensions and recorded. Asbestiform minerals of amphiboles, namely, grunerite, riebeckite, anthophyllite, tremolite and actinolite, and chrysotile (serpentine group) fibers, were recorded in the present study with fiber content calculated as millions of fibers per gram dry tissue. Non-asbestos mineral fibers, including aluminum silicate/mullite, kaolin, talc, muscovite, iron, silica, aluminum, and other metals, were recorded as per the analysis. At least 100 structures were analyzed or multiple grid openings. Analytic data were evaluated for all fiber lengths and for structures >5 μm. Data were contextualized against control populations of persons with no disease and no known exposure (other than ambient air breathing) or against control populations of persons with established pathologic asbestosis (asbestosis range controls).

### 2.3 Statistical methods

A Wilcoxon signed-rank test was used to assess whether the median of the paired differences between AB counts in FF and PE was significantly different from zero. Differences in paired samples were also analyzed with a Bland–Altman plot. The same tests were applied to analyze the paired differences between AAF counts in FF and in PE. To meet the assumption of normality of differences between paired samples in our dataset, base 10 was used for all logarithmic transformations. Estimated *p*-values of < 0.05 were considered to be statistically significant. Paired samples were not included in statistical tests if one or both of the results was below the limit of detection (LOD). All calculations and figures were produced using the Matplotlib, NumPy, pyCompare, and SciPy packages on a Python 3.11.7 environment.

## 3 Results

The first screening of the data showed that the results from PE are more often below the limit of detection (LOD) than the results from FF (Table 1). For AB analysis, 18 PE and 7 FF did not yield a result. For four paired samples, AB counts were below the LOD in both materials. Of the Zurich asbestos fiber analysis, 35 PE and 18 FF results were below the LOD. The results from 14 of these pairs were below the LOD in both materials. The cases submitted to the laboratory in Zurich comprised subjects with known as well as unknown or questionable exposure to asbestos. For the Cardiff samples, 35 PE and 12 FF did not yield amphibole asbestos fiber results. All Cardiff cases were submitted to investigate asbestos-related diseases in the absence of any known exposure. The results were below the LOD for the analysis in both materials in 10 paired samples. To this end, the analyses were consistent with an absence of any known significant occupational/domestic/environmental exposure.

**Table 1:** Number of analysis results below the limit of detection (LOD), listed according to type of analysis (AB, asbestos bodies; AAF, amphibole asbestos fibers) and material (FF, formalin-fixed tissue; PE, paraffin-embedded tissue).

Pairs without data		< LOD	
Zurich AB	FF	7	9%
	PE	18	24%
	FF + PE	4	5%
Zurich AAF	FF	18	24%
	PE	35	47%
	FF + PE	14	19%
Cardiff AAF	FF	12	26%
	PE	35	83%
	FF + PE	10	24%

FF + PE < LOD is an overlap of the other two groups, and therefore, the numbers are also incorporated in the other two groups.

During analysis, it was observed that PE samples had varying amounts of paraffin. This makes the comparative analysis more difficult as AB and AAF could be hidden underneath residual paraffin debris.

### 3.1 Asbestos body counts (Zurich laboratory)

**Figure 1:**
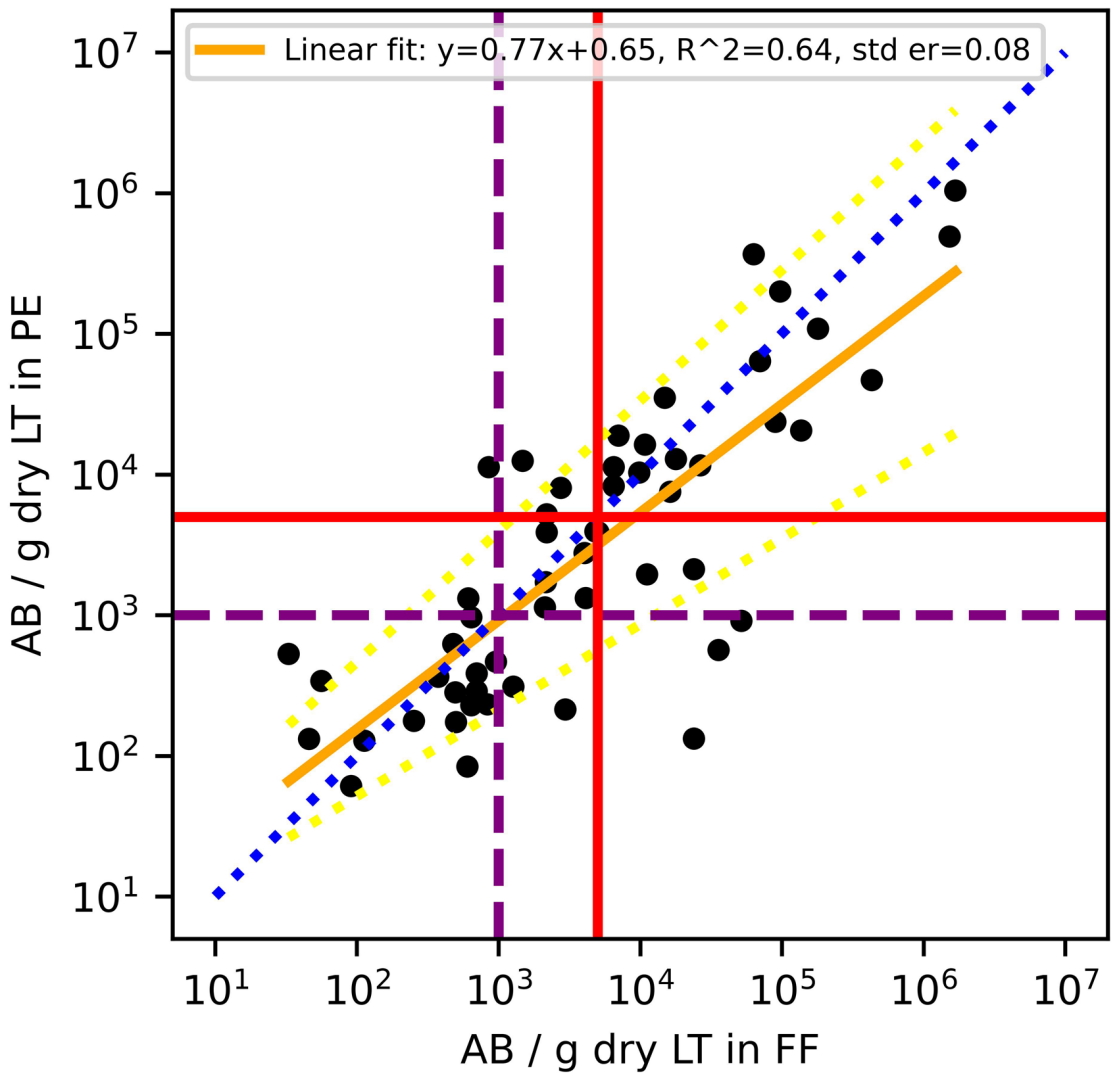
Comparison of asbestos body (AB) counts in formalin-fixed (FF) lung tissues and paraffin-embedded (PE) lung tissue of the same patients. According to the Helsinki criteria, 1,000 AB/g dry LT (purple dashed line) indicates a high probability of exposure to asbestos dust, and 5,000 AB/g dry LT (red solid line) is related to a 2-fold risk of developing lung cancer. The blue dotted line is the line of equality, the yellow solid line is the linear regression fit of log_10_ transformed residuals, and the dashed yellow lines are the 95% confidence interval of the regression.

**Figure 2:**
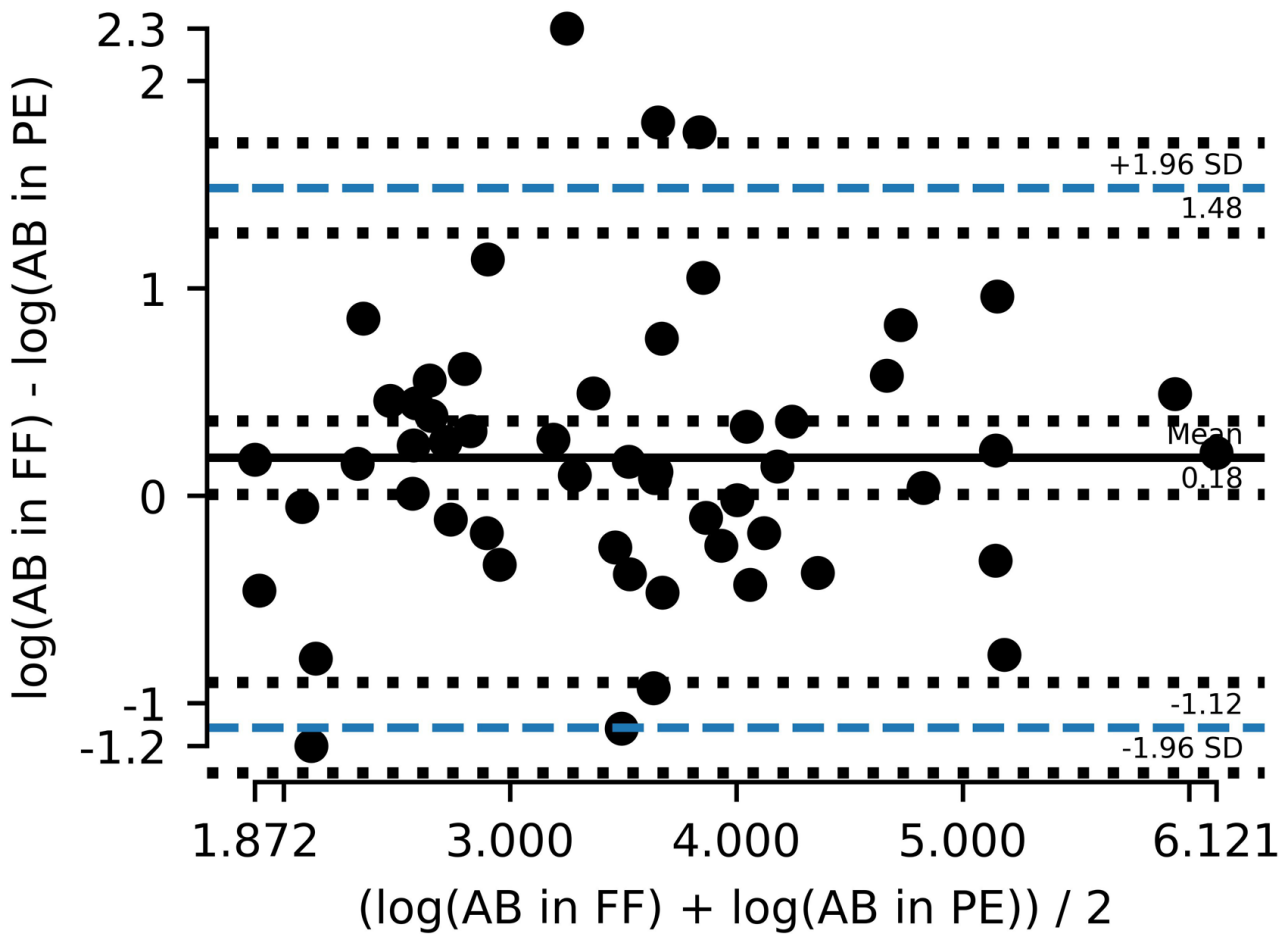
Bland–Altman plot showing the mean difference between asbestos body (AB) counts in formalin-fixed (FF) lung tissue and paraffin-embedded (PE) tissue and the 95% limits of agreement (dashed blue line). Counts are logarithmically transformed to meet assumptions of normality.

Out of the 75 paired samples from Zurich, 54 had AB counts above the LOD. Cardiff data did not include results on AB. As illustrated in Figure 1, the results of log_10_ transformed AB in FF counts plotted against AB in PE counts tended to be below the line of equality, suggesting that AB counts in PE tend to be lower than AB counts in FF. Although the paired differences between counted AB in PE and counted AB in FF were not significantly different from 0 (*p* = 0.06), there is evidence to suggest that the results between the two tests are not completely equivalent. On a Bland–Altman plot, the mean paired log_10_ difference between AB counts in FF and in PE (Figure 2) is 0.18 (*p*-value = 0.047, Table 2), also suggesting that counts of AB in PE may tend to be lower than those of AB in FF. Linear regression gives a fit with a slope of 0.77 (*R*^2^ = 0.64).

**Table 2:** Results from statistical analysis (Bland–Altman and linear regression).

Parameters	AB	AAF Zurich	AAF total
Bland–Altman analysis			
*t*	2.03	2.63	2.61
df	53	35	40
Bias	0.18	0.32	0.31
Upper 95% CI	1.48	1.76	1.80
Lower 95% CI	−1.12	−1.12	−1.18
*p*-value	0.047	0.013	0.013
Linear regression			
Intercept	0.65	1.33	1.56
Slope	0.77	0.74	0.71
*R* ^2^	0.64	0.51	0.51

### 3.2 Amphibole asbestos fiber counts

**Figure 3:**
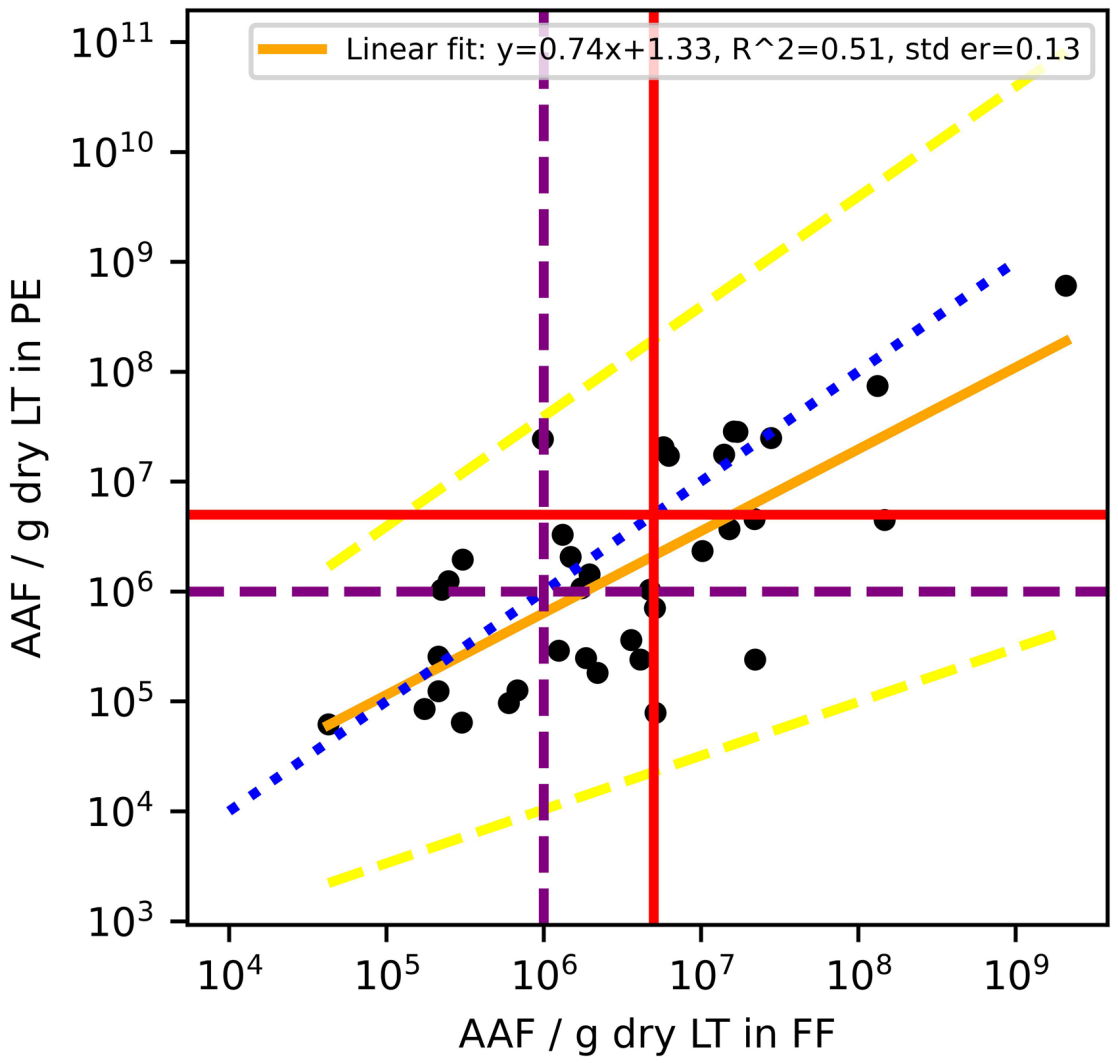
Amphibole asbestos fiber (AAF) counts in formalin-fixed (FF) lung tissue and paraffin blocks (PE) of the same patients. According to the Helsinki criteria, 1 Mio amphibole fibers (>1 μm)/g dry LT (purple dashed line) indicate a high probability of exposure to asbestos dust, and 5 Mio amphibole fibers (>1 μm)/g dry LT (red solid line) are related to a 2-fold risk of developing lung cancer. The blue dotted line is the line of equality, the yellow solid line is the linear regression fit of log_10_ transformed residuals, and the dashed yellow lines are the 95% confidence interval of the regression.

**Figure 4:**
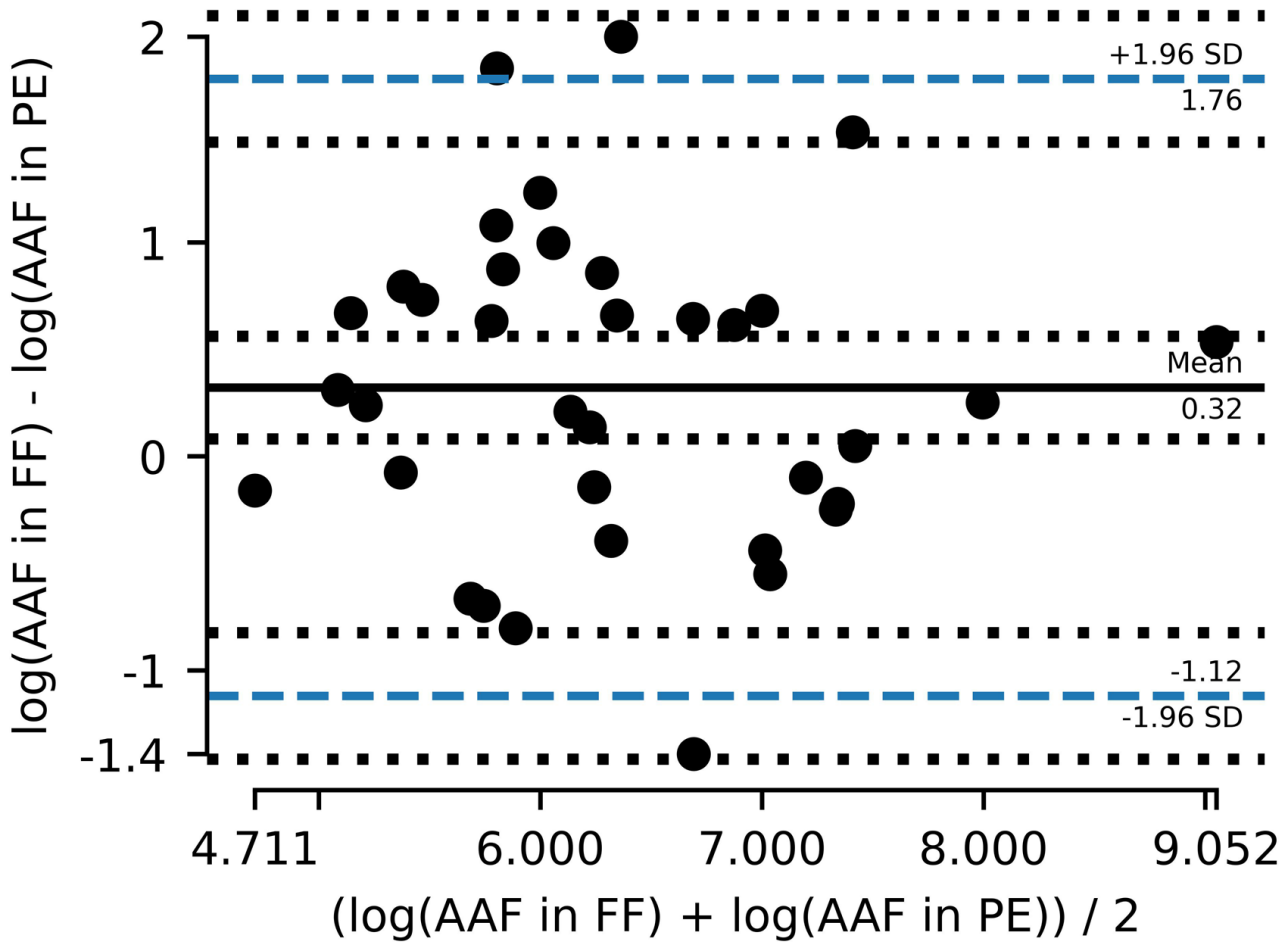
Bland–Altman plot showing the mean difference between amphibole asbestos fiber (AAF) counts in formalin-fixed (FF) lung tissue and paraffin-embedded (PE) tissue and the 95% limits of agreement (dashed blue line). Counts are logarithmically transformed to meet assumptions of normality.

A total of 36 out of the 75 paired samples from Zurich were available to evaluate AAF counts. The remaining pairs had either one or both values below LOD. Of the evaluated pairs, ~62% of PE samples had lower AAF counts than the corresponding FF samples (Figure 3), and the mean of paired differences between the two was statistically different from one another (*p* = 0.016). Furthermore, the Bland–Altman analysis for AAF counts showed an even greater deviation from 0, with the log_10_ transformed counts showing a mean difference of 0.32 (Table 2), again suggesting that AAF in PE tend to be lower than those in FF (Figure 4). Linear regression gives a fit with a slope of 0.74 (*R*^2^ = 0.51).

**Figure 5:**
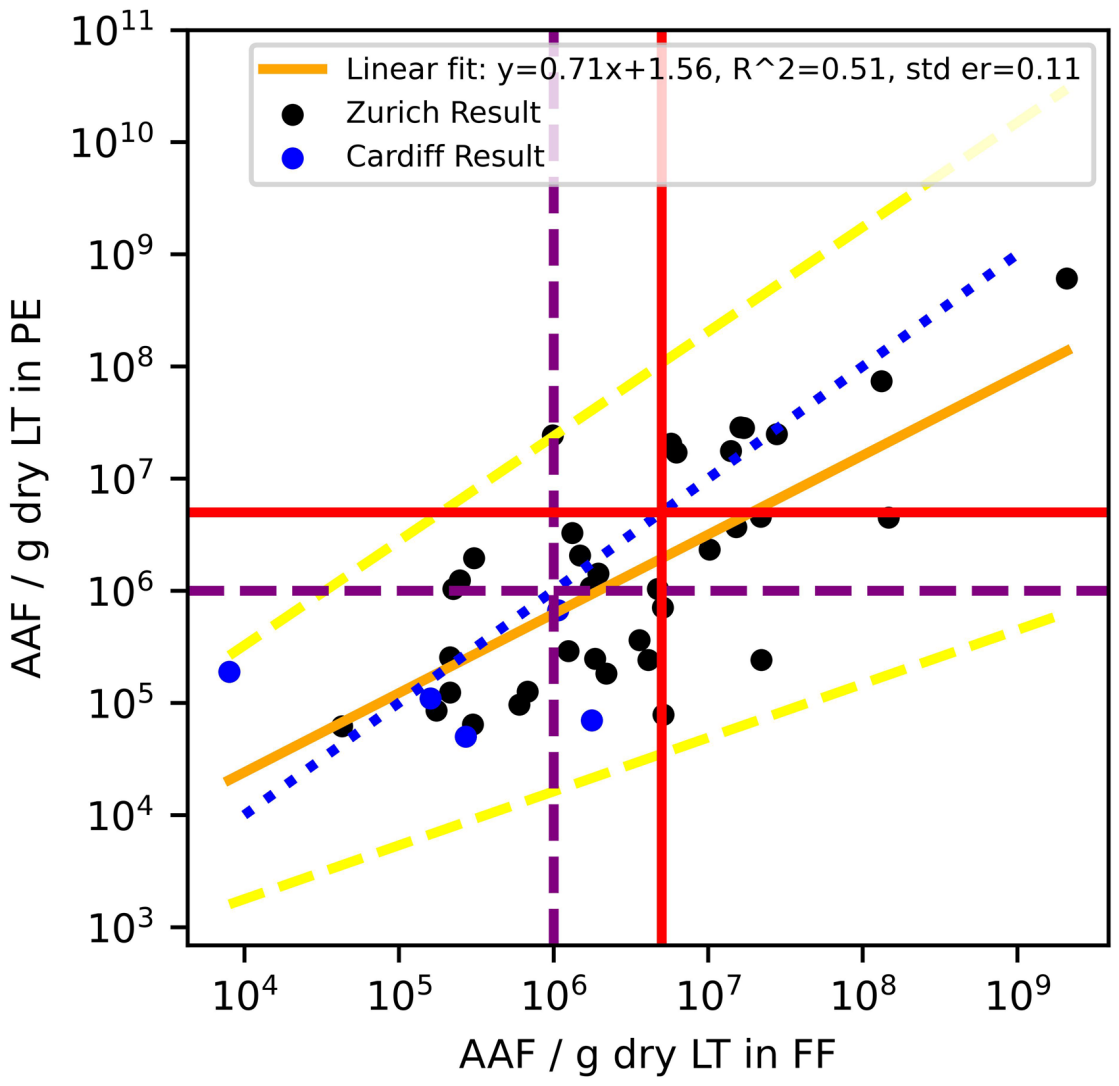
Combined data Zurich (black) and Cardiff (blue). Amphibole asbestos fiber (AAF) counts in formalin-fixed (FF) lung tissue and paraffin blocks (PE) of the same patients. According to the Helsinki criteria, 1 Mio amphibole fibers (>1 μm)/g dry LT (purple dashed line) indicate a high probability of exposure to asbestos dust, and 5 Mio amphibole fibers (>1 μm)/g dry LT (red solid line) are related to a 2-fold risk of developing lung cancer. The blue dotted line is the line of equality, the yellow solid line is the linear regression fit of log_10_ transformed residuals, and the dashed yellow lines are the 95% confidence interval of the regression.

**Figure 6:**
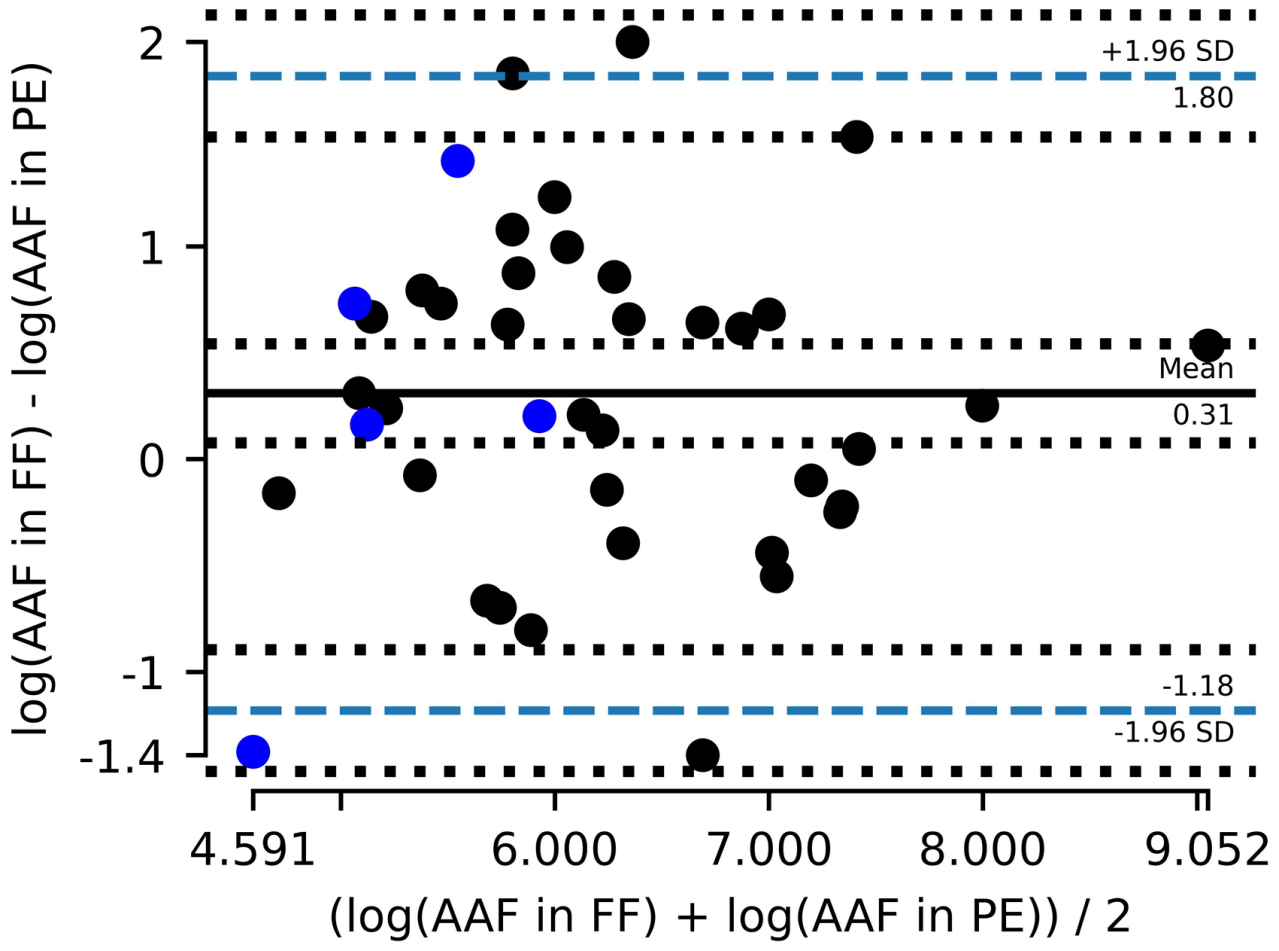
Combined data Zurich (gray) and Cardiff (blue). Bland–Altman plot showing the mean difference between amphibole asbestos fiber (AAF) counts in formalin-fixed (FF) lung tissue and paraffin-embedded (PE) tissue and the 95% limits of agreement (dashed blue line). Counts are logarithmically transformed to meet assumptions of normality.

Including the Cardiff data (5 out of 42, Figure 5) increased the normality of log_10_ transformed residuals (*p* = 0.9 –> 0.95) and had a Bland–Altman mean of 0.31 (Figure 6). The slope of the linear regression decreased to 0.71 (*R*^2^ = 0.51).

## 4 Discussion

Mineral fiber analysis has a central role in the pathologic evaluation of an individual’s cumulative asbestos exposure. Lung amphibole asbestos fiber content is one of the most important metrics in determining mesothelioma and lung cancer risk and is used in determining asbestos causation in these cancers and in cases of diffuse lung fibrosis (asbestosis). There are numerous correlative pathologic and mineralogic studies that show that mesothelioma, lung cancer, and grades of lung fibrosis correlate with retained amphibole asbestos fiber content in the lung but do not correlate with retained chrysotile due to its low biopersistence (6, 14–18). Mineral analysis represents the only method of determining an individual’s retained respirable fraction of asbestos following exposure, and this direct method of fiber content measurement contrasts with indirect subjective assessments determined by clinical methods, occupational history, or industrial hygiene dose reconstruction (19, 20).

Mineral analytic laboratories are required to establish control reference ranges for non-occupationally exposed subjects without disease, as well as individuals with disease (e.g., asbestosis), fulfilling the College of American Pathologists-Pulmonary Pathology Society guidelines (8) in the setting of establishing a laboratory ‘asbestosis range’. This is in keeping with the Helsinki criteria (19). These specific intralaboratory ‘control ranges’ allow scientists to contextualize the significance of an individual test result and exposure conducted at that site against established reference ranges. While there are different methods of mineral analysis with varied sensitivity and specificity, TEM, as conducted in this study, yields quantitative and qualitative data and represents the most sensitive means of evaluating mineral content in biological samples.

### 4.1 Key findings

With respect to AB and AAF counts conducted in corresponding FF and PE, respectively, this study identified that numeric values in the paired samples processed differently do not yield identical results, with PE samples having lower numeric values than those from FF samples in >60% of the analyses (62% for AB counts; 64% for AAF counts). Statistical analysis (paired differences and Bland–Altman test, Table 2) showed that the difference is statistically significant for AAF. In Switzerland, asbestos burden analysis results of submitted cases are benchmarked against the published Helsinki criteria (19, 20). In the Zurich data, this would therefore lead to different classifications according to the Helsinki criteria for a 2-fold risk of developing lung cancer in 11% (6 out of 54) of the cases using AB results and 19% (7 out of 36) using AAF content. In the Cardiff laboratory which established internal controls for background cases and asbestosis, the differences do not impact overall interpretation.

### 4.2 Sources of variability

It is important to consider how AB and AAF counts may yield variable results in biological samples. Asbestos body counts correlate closely with retained commercial amphibole asbestos fibers and far less with non-commercial amphibole asbestos and chrysotile fibers (21). First, there are recognized anatomic variations in the distribution of AB, AAF, and chrysotile fibers in the lung. The heterogeneous distribution of asbestos in the lung has been described by various authors (5, 22–24). Churg and Wood (25) reported high variability for AB (up to 5.4-fold) and total asbestos fibers (up to 7-fold) in adjacent small tissue samples of 1 cm^3^ each. This is in contrast with their repeated measurements of mineral standard materials that only showed differences up to 1.5-fold. To even out these heterogeneities, in the Swiss laboratory, relatively large samples of FF (average sample size: 3.91 ± 0.96 g for AB and 4.93 ± 0.85 g for AAF) tissue were used. The sample size of PE is much smaller (AB: 0.24 ± 0.12 g; AAF: 0.46 ± 0.23 g), and thus, the heterogeneity of lung samples and variation of structures present may account for some of the differing results. In the UK laboratory, only post-mortem samples were used, with optimal three ~2 cm^3^ pieces sampled from the apex upper lobe, apex lower lobe, and lung base, collectively in a ‘pooled’ analysis; or corresponding tissue of similar size processed into paraffin blocks, as set out in the Good Practice Guidelines for Post-mortem examination in suspected industrial disease deaths including asbestos (9). This study is subject to random error that occurs in the distribution of AB and AAF across the lung, but using multiple pooled samples reduces this random error. In addition, the researchers consider that systematic error borne of sampling inappropriate biologic tissue is far less likely to account for the observed differences as macroscopic and microscopic quality control checks are performed to prevent such an outcome.

It is also important to consider technical bases for AB and AAF variation in the different biologic tissues. Specimen preparation, including processes of fixation, tissue storage, and fiber extraction methods (digestion or ashing, solvent usage), may all impact fiber yield and fiber size content (24, 26, 27). In addition, paraffin wax/water baths may be contaminated with asbestos fibers. Low-temperature ashing methods have been shown to result in fiber fragmentation and elevated fiber counts (26, 27). In contrast, wet digestion techniques can result in fiber loss (24). Sample preparation methods have been adapted to minimize fiber loss, but compared to FF, PE undergoes several washing steps during dehydration before impregnation with paraffin and several more washing steps for dewaxing. In addition, the tissue embedded in paraffin is cut into very thin slices that have a large surface area for a small volume. Incomplete organic tissue digestion, sometimes only evident on grid analysis, may result in fiber aggregation. The low-power filter examination of the grids in this study ensured uniform particulate dispersion.

### 4.3 Limitations and recommendations

In seeking to address the shortfalls that may arise in some fiber counts, one approach is the use of a correction factor to more reliably translate numeric values for AB and AAF counts determined from paraffin-embedded alone to formalin-fixed lung tissue. However, in the case of our observations, the paired differences of AB and AAF count data are not normally distributed and, therefore, need to be log_10_ transformed for statistical analysis. Any correction factor in this case would give predicted values in log_10_ space, which then would have to be transformed into real space. These back extrapolations are technically possible but result in reduced precision of predictions, hindering predictive value. In addition, the influence a few values from another laboratory have on the linear regression suggests that each laboratory would need to establish its own linear regression. For this, a fair number of samples would be needed with a large range of asbestos content. Furthermore, both AB and AAF regression effect sizes were moderate, as shown in *R*^2^ values in Figures 1, 3, 5, indicating a moderate relationship between the two paired measurements. Therefore, using them to directly predict resultant AB and AAF counts may lead to imprecise results.

While the numeric values for AB and AAF in lung tissue obtained using either FF or PE tissue may not be identical, a closer analysis of the etiologic significance shows that only in a small minority of cases does the difference in count potentially impact the significance of the etiologic conclusion. The researchers note that the Swiss and UK analytic laboratories differ in the significance they assign to mineralogic causal attribution criteria for lung cancer as set out in the Helsinki criteria. In Switzerland, it is general practice to benchmark both AB and AAF (fiber size dependent) together with occupational exposure estimates (fiber-years) against the stated strict numeric criteria established in the 1997 Helsinki criteria (28) for AB and AAF. The etiologic significance of these values is derived from different European laboratories. In contrast, the UK laboratory benchmarks individual results against control populations from within the same laboratory, including subjects without disease and those with asbestosis. This involves using an internally evaluated ‘asbestosis range’ for lung cancer and lung fibrosis attribution cases and controls within the laboratory, alongside pathologic assessment of lung tissue for asbestosis. The UK laboratory establishes its own reference ranges, an approach also adopted by others (29, 30). This approach diminishes the significance of the differing results obtained from FF and PE samples.

Mineral analysis is advocated for use in specialized laboratories in which reference ranges have been established. Etiologic inferences without appropriate internally validated benchmark controls may result in erroneous conclusions. Differences in methodology (FF/PE) often yield different numeric results; however, in the wider context, these rarely impact the etiologic conclusion. In cases where no AB or AAF content is detected by either preparation, no causal attribution to asbestos can be made on mineralogic grounds. Control reference ranges established using one methodology do not necessarily hold the same significance in another laboratory using another methodology. For this reason, following histologic confirmation, the asbestosis range remains the single most useful mineralogic criterion, as advocated by the Consensus report and Helsinki criteria for establishing lung cancer and fibrosis causation, since it does not rely on transferring disease causation significance between laboratories (20, 28).

## 5 Conclusion

Mineral analysis remains a key tool in assessing prior exposures to asbestos, with mesothelioma, lung cancer, and lung fibrosis/asbestosis correlating closely with retained AB and AAF content in lung tissue. Mineral analysis remains the only objective marker of the respirable fraction of an airborne exposure. This requires suitable formalin-fixed lung tissue, preferably from multiple sites in a ‘pooled’ analysis to yield the most representative results for an individual’s prior exposure. This study highlights that numeric values for fiber yield may vary between samples. Pathologists should be aware of how tissue sampling impacts mineral analytical results. In legal settings, it is a matter for the Court to determine where reliance be placed when claimed exposures, determined by either clinical history—patient, co-worker, or relative interviews, and/or industrial hygienist dose-reconstruction estimates are disparate from each other and/or from mineral analytic results in lung burdens.

## Data Availability

The raw data supporting the conclusions of this article will be made available by the authors, without undue reservation.
